# Evaluation of a Parental Questionnaire to Identify Atopic Dermatitis in Infants and Preschool Children

**DOI:** 10.1155/2012/945617

**Published:** 2012-02-14

**Authors:** Laura B. von Kobyletzki, Staffan Janson, Mikael Hasselgren, Carl-Gustaf Bornehag, Åke Svensson

**Affiliations:** ^1^Department of Dermatology, Institute of Clinical Research in Malmö, Skåne University Hospital, Lund University, 205 02 Malmö, Sweden; ^2^Department of Public Health Sciences, Karlstad University, 651 88 Karlstad, Sweden; ^3^School of Medicine, Örebro University, 701 82 Örebro, Sweden

## Abstract

*Aim*. To develop and validate a questionnaire for detecting atopic dermatitis in infants and small children from the age of 2 months. *Methods*. Parents to 60 children answered a written questionnaire prior to a physical examination and individual semistructured interview. Qualitative and quantitative analyses of validity, sensitivity, specificity, and predictive values of the questionnaire were performed. *Results*. A total of 27 girls and 33 boys, aged 2 to 71 months, 35 with and 25 without physician-diagnosed eczema, participated. Validation of the questionnaire by comparisons with physicians' diagnoses showed a sensitivity of 0.91 (95% CI 0.77–0.98) and a specificity of 1 (95% CI 0.86–1). *Conclusions*. Three questions in a parental questionnaire were sufficient for diagnosing eczema in infants and small children.

## 1. Introduction

 Atopic dermatitis affects 15 to 20% of preschool children in western countries [1–4]. The lifetime prevalence of eczema in children aged 1–4 years in Värmland county, Sweden, was assessed in 2000 in the cohort study Dampness in Building and Health to be 22% [5]. The burden of disease has been assessed to be at least comparable to other chronic illnesses, such as diabetes or neurological disorders [6–8]. For population-based, epidemiological investigations, parental written questionnaires detecting eczema during childhood are advantageous. However, current questionnaires have been developed for older children and adults [9]. Given that in small children atopic dermatitis is the most common inflammatory disease, the deficiency of validated questionnaires diagnosing eczema in very young children is noteworthy [10]. A questionnaire should be suitable to the general population and therefore evaluated in such a setting. Furthermore, it should be applicable to eczema with different severity categories. A new questionnaire detecting atopic dermatitis in young children could probably be based on previous tools for older children.

For the current study, a questionnaire was based on the existing ISAAC (International Study of Asthma and Allergies in Childhood) questions for school children [9]. The aim of the current study was to estimate the diagnostic precision of the children's eczema questionnaire compared to physicians' clinical diagnoses as the gold standard in preschool children from the age of two months. Further, we evaluated whether the parents considered the questions to be understandable and suitable for diagnosing eczema in this age group.

## 2. Methods

### 2.1. Study Design and Procedures

**Figure 1:**
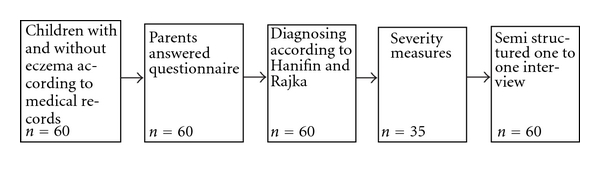
Study procedures.

A case control design similar to that used by Williams et al. in his validation study on atopic dermatitis was chosen [11]. As seen in Figure 1, 35 children with eczema and 24 without eczema according to medical records and aged two months to six years were recruited consecutively from a preventive care register in Arvika, Värmland County, Sweden. These 60 children's parents answered a questionnaire on eczema (the questionnaire to be evaluated). Then, these 60 children were examined by a physician. Physician diagnosis, based on the presence of at least three major and three minor criteria of Hanifin and Rajka's diagnostic criteria [12, 13], was used as the gold standard to validate the questionnaire. The physician was blinded to the answers of the questionnaire. In clinical eczema cases, severity was measured with the SCORing Atopic Dermatitis (SCORAD) index [14]. Furthermore, all parents answered a background questionnaire.

The qualitative part of the investigation was carried out by means of a semistructured, one-to-one interview with the parents. The interview started with a general discussion regarding the understanding of the eczema questions and relevance of named issues on infants' and preschool children's eczema. Each interview was conducted by the same physician, the first author of this paper, to ensure consistency. The interview was transcribed and approved by the responders.

The regional ethical committee in Uppsala approved the protocol for the study, Dnr-C2007/41. Written consent was obtained 2 weeks prior to study start. 

### 2.2. Study Population

Inclusion criteria for index cases were eczema, treated either with emollients or topical glucocorticoids. Inclusion criteria for controls were an age of two month to six years and no eczema. Exclusion criteria were eczema treated with systemic treatment, other chronic diseases such as diabetes, acute infections, or if parents did not understand or speak Swedish fluently. A power analysis showed we would need 60 children to assess sensitivity and specificity with a power of 0.8 and alpha 0.05 [15]. A total of 62 children were invited to participate, but there were two denials. Data on 60 children could be analysed.

### 2.3. How the Parental Questionnaire was Constructed

Atopic dermatitis was defined by Hanifin and Rajka as *pruritic*, *chronically relapsing dermatitis*, with typical *features and distribution* [13]. Our questionnaire was constructed to discriminate whether these essential signs of eczema existed in each child or not. The questions we used for this were derived from the ISAAC protocol, which was developed for school children. We used the main outcome measure of the ISAAC protocol, “Has your child ever had an itchy rash which was coming and going for at least 6 months?”, and the question, which is an additional question in the ISAAC protocol “Has this itchy rash at any time affected any of the following places: the folds of the elbows, behind the knees, in front of the ankles, under the buttocks, or around the neck, ears or eyes?”. We adopted the questions to the age group in our study. The main adoption was that we divided the main outcome measure of the ISAAC protocol into two parts, thus we asked for *chronic relapsing dermatitis* and* itch *in two separate questions. Further, we extended the list of involved sites, since more sites can be involved in younger children. Involved sites were in agreement with Halkjaer's description of eczema distribution in young children [16], and face validity was confirmed by comparing our criteria with the UK diagnostic criteria for atopic eczema [11].

The three key questions are as follows.

- Does your child have or has your child had a *red rash/eczema which can come and go*?
- If Yes, has this caused *itching or scratching*?
- Has this red rash/eczema affected any of the *following areas* (during the last week): Around the eyes, ears, scalp, cheeks, forehead, neck, trunk, folds of the elbows/behind the knees, wrist or ankle, outer arms/legs?

The children had to answer “yes” in each of the three questions to be classified as a questionnaire diagnosed eczema.

## 3. Analysis

### 3.1. Quantitative Analyses

 A questionnaire-diagnosed eczema was compared to a physician's diagnosis which was the gold standard. Sensitivity and specificity as well as the positive predictive value (PPV) and negative predictive value (NPV) of the sample population and stratified age groups were assessed.

PPV was calculated as (prevalence × sensitivity)/(prevalence × sensitivity + (1 − prevalence) × (1 − specificity)) [17]. NPV was calculated as (1 − prevalence)/((1 − prevalence) + prevalence × (1 − sensitivity)/specificity) [18]. There was no missing data. Statistical analysis was performed with Stata tm 10.1 Statistics Data Analysis.

### 3.2. Qualitative Analysis

 Data was analysed according to content [19]. The most commonly mentioned and coded topics formed the basis of further defining themes and patterns. Interpretations were compared to the original data for internal corroboration or disconfirmation. Findings were summarized regarding the understanding of the eczema concept and relevance of the diagnostic questions.

## 4. Results

### 4.1. Patient Characteristics

Eczema occurred in 35 children, who reported dry skin twice as much as children without eczema (Table 1). Both children with and without atopic dermatitis manifested other skin disorders at the same rate as miliaria, haemangioma, keratosis pilaris, and melanocytic naevi. In the eczema group, eleven (33%) children awoke several times per week, and 2 (6%) awoke occasionally because of skin symptoms according to parental reports. The treatment of the children with eczema consisted of emollients and topical glucocorticoids. The latter had been used in only two cases during the last week. No other active treatment was reported. Further characteristics of the children are shown in Table 1.

### 4.2. Validation of the Used Questionnaire

A combination of affirmative answers to all three key questions, rash, itch, and location, predicted clinical eczema with good sensitivity 0.91 (95% CI 0.77 to 0.98) and specificity 1 (95% CI 0.86 to 1).

Predictive values were PPV = 1 and NPV = 0.976. Diagnostic accuracy by age groups was similar (Table 2).

### 4.3. Results of the Qualitative Study

Most parents judged the questions to be understandable and suitable—“*even if I do not deal much with asthma and allergy.*” According to the parents, the questionnaire covered all the important facts regarding the eczema diagnosis. However, disease terms were regarded to be difficult; “*eczema is too strong an expression to answer yes to*” whereas questions concerning symptoms were thought to be easily answered—“*easy, one can already see itching at the age of 6 months.*” 

Of 35 parents to children with eczema, 21 expressed feelings of stress and worry about their child's health state and felt responsible for it. Four parents reported not wishing their children to be diagnosed with eczema at all because of fear of cortisone treatment. Itching and awakening due to itching were regarded as the most burdensome symptoms.

## 5. Discussion

With three key questions, atopic dermatitis in preschool children can be identified via a parental questionnaire. The high sensitivity and specificity indicate that this questionnaire is a valuable diagnostic tool. Validated eczema questionnaires have been available for school children only. But the incidence of eczema is highest in preschool children. Our questionnaire provides a feasible diagnostic tool with high diagnostic precision, which can add to the field of epidemiological research in early childhood eczema.

The semistructured interview confirmed high face and content validity, which might be one reason for the high proportion of children with detectable eczema. The PPV of the questionnaire is the proportion of children with a positive result who actually had physician-diagnosed eczema. Predictive values are, however, related to the prevalence of eczema in the population, which could not be assessed with this study design. The high prevalence of eczema, 22%, in the population was derived from data from the same county [5]. The results suggested that the risk of diagnosing eczema in healthy infants and small children was low. Sensitivity measures the proportion of actual positives, which are correctly identified as such. Even the measured sensitivity was high, indicating that not many eczema diagnoses were missed. The questionnaire did not suggest healthy children to be diseased, as shown by the high specificity.

It is important to estimate the severity of eczema, both for the description of the study population and for an assessment of generalizability. A score combining an assessment of disease extent with clinical features, duration plus intensity (SCORAD) was used [14]. Based on this assessment, participating children showed mild-to-severe eczema that allows applying the questionnaire in all severity groups.

The strength of the study is the population-based setting, as the questionnaire is intended to be used in a similar setting. Because all children were registered in a preventive care register and almost all invited children participated, it is unlikely that selection bias occurred, even though the sample was not randomly selected. Assessing the diagnostic accuracy of the diagnostic test on a sample of the general preschool child population allows generalizing results. The narrow confidence intervals suggest that the sample size was adequate and that estimates of the population value are within a reasonable range. Compared to a standardized skin examination protocol, the ISAAC questionnaire performed well in predicting eczema prevalence at the population level. However, on an individual level, a high proportion of flexural eczema was not confirmed by skin examination [20]. A study from Brazil tested the ISAAC questionnaire against a gold standard. Even here a high proportion of the ISAAC cases were false and many physician-diagnosed cases were missed [21]. In contrast, our study showed good performance on an individual level. This does not mean that our questionnaire is to be preferred but that the questions which we adopted to our cultural environment might be useful in Sweden as shown by the validation against a gold standard. In different linguistic and cultural contexts, an adoption of the questions and new validation should be considered [22]. It is for example possible that the questionnaire performs differently in populations with less access to health care.

## 6. Conclusions

This study makes a contribution in solving diagnostic problems where there has been a lack of diagnostic questionnaires for small children. The questionnaire can be used in a population-based setting and across different severity groups of eczema. It is, however, important to take cultural aspects into account.

## Figures and Tables

**Table 1:** Baseline characteristics of children with and without eczema due to specific criteria.

	Patients' diagnose status		
	Eczema^a^	Noneczema	*Total*

Number, *n* (%)	35 (58.3)	25 (41.7)	*60 *(*100*)

Age (month)			
Mean (SD)	25.96 (±19.1)	25.68 (±18.3)	*25.84 *(±*18.6*)
Median (range)	18.0 (4–66)	19.0 (2–60)	*19.0 *(*2–66*)

Sex, *n* (%)			
Female	19 (54.3)	8 (32.0)	*27 *(*45.0*)
Male	16 (45.7)	17 (68.0)	*33 *(*55.0*)

Heredity, *n* (%)			
Yes	27 (77.1)	15 (60.0)	*42 *(*70.0*)
No	8 (22.9)	10 (40.0)	*18 *(*30.0*)

Dry skin, *n* (%)			
Yes	27 (81.8)*	6 (18.2)*	*35 *(*58.3*)
No	8 (29.63)*	19 (70.4)*	*25 *(*41.7*)

SCORAD Score^b^, *n* (%)			
Severe	6 (17.1)		
Moderate	15 (42.9)		
Mild	14 (40.0)		

^a^Physician diagnose, based on Hanifin and Rajka's criteria.

^b^Eczema severity assessed with SCORing Atopic Dermatitis.

*P (Pearson chi2(1)) <0.05.

**Table 2:** Validation of the used questionnaire by comparison of parental reported eczema with physician diagnose of the children by age group.

Patients' age (month)	Psychometric measures			
	Sensitivity (95% CI)	Specificity (95% CI)	Positive predictive value	Negative predictive value
0 to 23	0.91 (0.70–0.99)	1 (0.81–1)^b^	1	0.97
≥24	0.93 (0.66–1.00)	1 (0.76–1)^b^	1	0.98
0 to 66 (total)	0.914 (0.77–0.98)	1 (0.89–1)^b^	1	0.98

^b^Single sided 95% CI.

## Abbreviations

SCORAD: SCORing Atopic Dermatitis
PPV: Positive predictive value
NPV: Negative predictive value.
